# Study of Abnormal Group Velocities in Flexural Metamaterials

**DOI:** 10.1038/s41598-019-50146-8

**Published:** 2019-09-27

**Authors:** Hong Woo Park, Joo Hwan Oh

**Affiliations:** School of Mechanical, Aerospace and Nuclear Engineering, Ulsan National Institute of Science and Technology, UNIST-gil 50, Eonyang-eup, Ulju-gun, Ulsan 44919 Korea

**Keywords:** Mechanical engineering, Acoustics

## Abstract

Generally, it has been known that the optical branch of a simple one-dimensional periodic structure has a negative group velocity at the first Brillouin zone due to the band-folding effect. However, the optical branch of the flexural wave in one-dimensional periodic structure doesn’t always have negative group velocity. The problem is that the condition whether the group velocity of the flexural optical branch is negative, positive or positive-negative has not been studied yet. In consequence, who try to achieve negative group velocity has suffered from trial-error process without an analytic guideline. In this paper, the analytic investigation for this abnormal behavior is carried out. In particular, we discovered that the group velocity of the optical branch in flexural metamaterials is determined by a simple condition expressed in terms of a stiffness ratio and inertia ratio of the metamaterial. To derive the analytic condition, an extended mass-spring system is used to calculate the wave dispersion relationship in flexural metamaterials. For the validation, various numerical simulations are carried out, including a dispersion curve calculation and three-dimensional wave simulation. The results studied in this paper are expected to provide new guidelines in designing flexural metamaterials to have desired wave dispersion curves.

## Introduction

Flexural metamaterial is an artificially-made elastic metamaterial that tailors flexural waves by periodically arranged sub-wavelength unit cells. By using previously known dynamics of metamaterials, such as the internal resonance phenomena^1–11^ or Bragg scattering^12–16^, numerous flexural metamaterials have been proposed^17–24^. Recent researches about flexural metamaterials focused on its unique characteristics^25,26^ which originate from the fact that flexural wave is governed by its own unique equation unlike other metamaterials^27^. For example, Lee *et al*.^25^ reported that flexural metamaterial accompanied with rotational resonance exhibits peculiar bandgap phenomena that cannot be explained with previously developed theories on metamaterial. Also, Oh *et al*.^26^ showed that a first-order wave mode could be flattened with zero rotational stiffness so that a broad, low-frequency bandgap can be obtained. However, despite the previous researches, unique wave characteristics of flexural metamaterials are still not fully understood.

**Figure 1:**
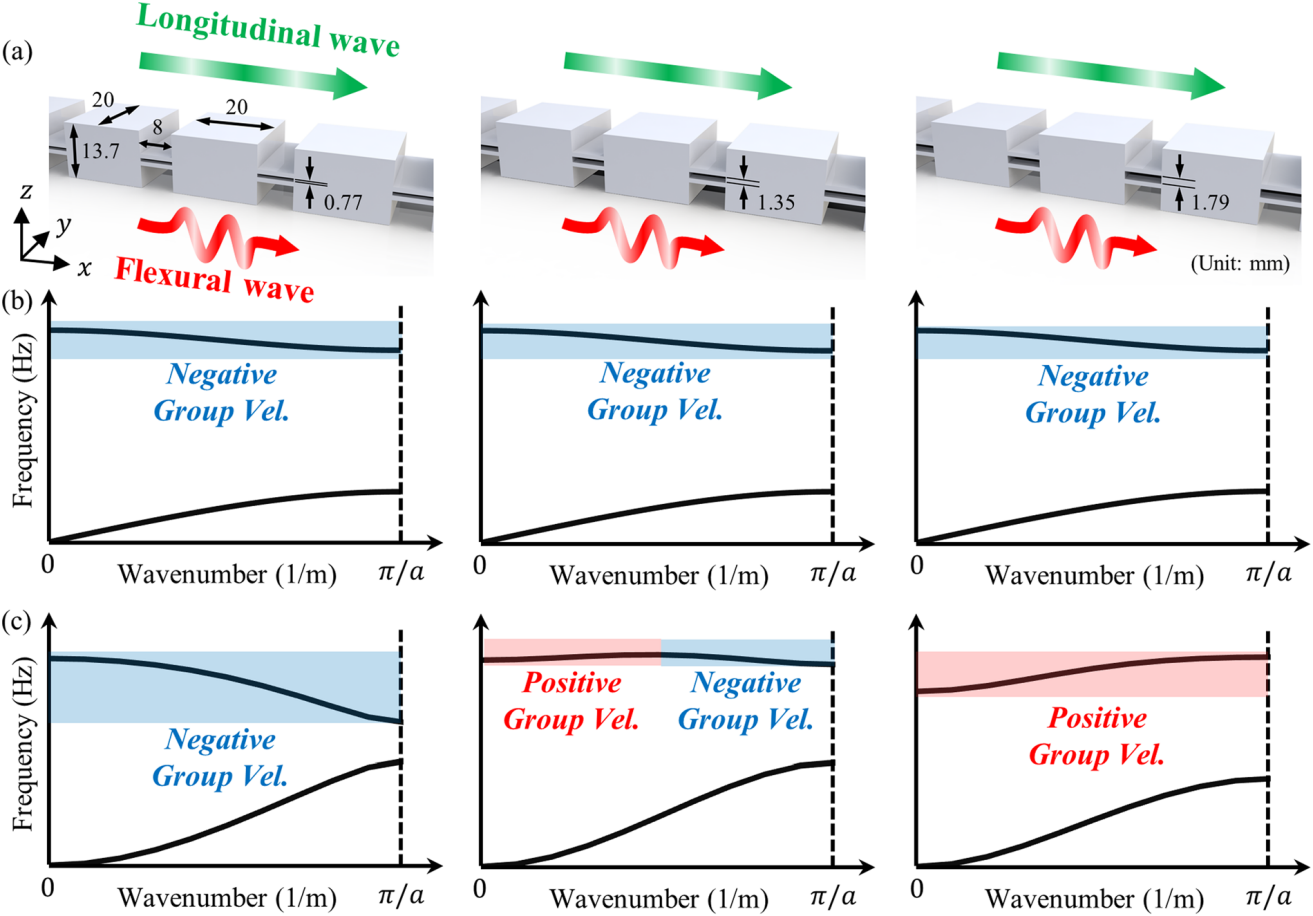
(**a**) Various simple one-dimensional periodic systems made by aluminum (thickness of the two connectors is 1 mm), (**b**) the dispersion curves for longitudinal waves show a negative group velocity on the optical branch, (**c**) the dispersion curves for flexural waves show negative, positive-negative and positive group velocity on the optical branch.

Here, we’d like to focus on the group velocity issue among the other unique characteristics of the flexural metamaterials. For better explanation about the issue, the dispersion curves of flexural wave mode are compared with the longitudinal wave mode as shown in Fig. 1. When the longitudinal wave propagates inside the periodic structures, shown in Fig. 1(a), the optical branch of the dispersion curve usually has a negative group velocity, as in Fig. 1(b), because it originates from the well-known band-folding at the Brillouin zone. (Here, we will use the term ‘acoustic branch’ and ‘optical branch’^28^ to describe the lower and upper wave dispersion branch, respectively. Also, the group velocity is defined in the first Brillouin zone^29^, following the general definition)^30,31^. However, this general understanding does not hold for flexural wave; the optical branch of the flexural metamaterial does not always have negative group velocity. For the flexural wave, the optical branch could have negative, positive or even positive-negative group velocity, as in Fig. 1(c). According to the previous researches on elastic metamaterials^30–33^, the optical branch with both positive-negative group velocity is usually observed if the material is two- or three-dimensional structure^30,31^, or if there exists any coupling between various elastic wave modes^32,33^. However, as can be seen in Fig. 1, none of the case belongs to the flexural wave’s group velocity issue - the group velocity issue is observed for even one-dimensional simple periodic structure where flexural wave mode is not coupled with any other wave modes such as longitudinal, shear or torsional wave modes. Because of this unique characteristic, previous research utilizing negative group velocity in periodic structures, such as negative refraction^30,31,34^ or wave-focusing^34,35^, cannot be directly applied to the flexural metamaterial. In consequence, there is currently no design guideline to utilize the optical branch in flexural metamaterials and they have been designed by trial-and-errors.

In this paper, we present an analytic investigation and background physics of the group velocity issue in the optical branch of flexural metamaterials. From the analytic approach, we found that there is a certain condition that determines whether the optical branch has purely positive, negative or both positive-negative group velocity. The condition can be simply expressed by the stiffness ratio (the ratio between shear and bending stiffness of the flexural metamaterial) and the inertia ratio (the ratio between mass and rotational inertia of the flexural metamaterial). From the study, one can easily design the flexural metamaterial to have negative, positive or positive-negative group velocities by adjusting the stiffness ratio and the inertia ratio. Also, we figured out the background physics that cause the group velocity issue, with the explanation why the issue is hardly observed in other metamaterials.

To analytically study the group velocity issue in optical branch of flexural metamaterials, we used the extended mass-spring system developed by Oh *et al*.^26^ for flexural metamaterials. The extended mass-spring system is based on the classical Timoshenko beam theory^26^. In the extended mass-spring system, each mass is assumed to have two degrees of freedom - the vertical and rotational motions - and the dispersive nature of flexural metamaterial can be precisely and easily studied. Therefore, there is no need to use the complicated guided wave theories or to extend the Kirchhoff plate theory to periodic systems to consider the flexural metamaterial. From the extended mass-spring system, analytic condition governing the group velocity in flexural metamaterial is derived. To support the findings from our analytic investigation, numerical validations are carried out by numerically calculating the wave dispersion curves for various flexural metamaterials. Also, three-dimensional wave analyses are carried out to show that the derived analytic conditions and related findings can be used for three-dimensional systems.

## Results

### Analytic condition for the group velocity issue of the optical branch

**Figure 2:**
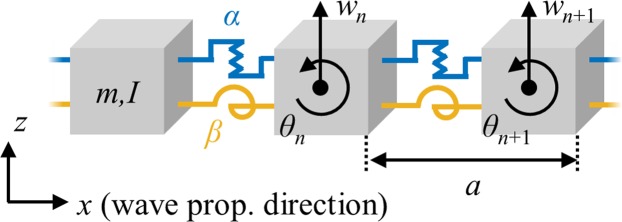
Extended mass-spring system for flexural metamaterials which is governed by vertical motion (*w*_*n*_) and rotational motion (*θ*_*n*_). The unit cell, arranged with periodicity (*a*), consists of mass (*m*), rotational inertia (*I*), linear spring (*α*) and rotational spring (*β*).

Unlike acoustic or other elastic wave modes, flexural waves are governed by different wave equations and are thus hard to be expressed in general mass-spring systems. Therefore, the extended mass-spring system shown in Fig. 2 was developed to consider the flexural wave with discrete mass-spring system^25,26^. Since this system is based on the classical Timoshenko beam theory, it cannot be used for very high frequency ranges. However, in current research, we consider the frequency range covering acoustic and optical branches, and the extended mass-spring system can precisely predict flexural wave motion at this frequency range^25,26^. In Fig. 2, the *n*^*th*^ unit cell with the periodicity of *a* consists of two kinds of inertias (mass *m* and rotational inertia *I*) and two kinds of springs (vertical linear spring *α* and rotational spring *β*) in order to consider two kinds of motions, vertical linear motion *w*_*n*_ and rotational motion *θ*_*n*_, respectively. With this system, the wave dispersion relationships can be obtained as (see the detailed derivation in Supplementary Material):

1 $$Im{\omega }^{4}+A\,(k){\omega }^{2}+B(k)=0$$

where *A*(*k*) and *B*(*k*) are defined as:

2a $$A(k)=2(\alpha I+\beta m)[\,\cos \,(ka)-1]-\frac{1}{2}\alpha m{a}^{2}[\,\cos \,(ka)+1]$$

2b $$B(k)=4\alpha \beta {[\cos (ka)-1]}^{2}$$

By using the quadratic formula, Eq. (1) can be solved with respect to *ω*^2^ as:

3a $${\omega }^{2}=\frac{-A(k)-\sqrt{A{(k)}^{2}-4ImB(k)}}{2Im}$$

or

3b $${\omega }^{2}=\frac{-A(k)+\sqrt{A{(k)}^{2}-4ImB(k)}}{2Im}$$

Equations (3a) and (3b) indicate that there are two different dispersion curves in the extended mass-spring system. Here, Eq. (3a) corresponds to the acoustic branch, while Eq. (3b) corresponds to the optical branch of the flexural wave in the periodic system.

To determine whether the optical branch has a positive or negative group velocity, the analytic equation for the group velocity of the optical branch should be considered. The equation for the group velocity can be achieved by differentiating the dispersion relation in Eq. (1) with wavenumber *k* as:

4 $$4Im{\omega }^{3}\frac{\partial \omega }{\partial k}+A^{\prime} (k){\omega }^{2}+2A(k)\omega \frac{\partial \omega }{\partial k}+B^{\prime} (k)=0$$

where *A*′(*k*) and *B*′(*k*) are defined as:

5a $$A^{\prime} (k)=\frac{\partial A(k)}{\partial k}=-\,[2(\alpha I+\beta m)-\frac{1}{2}\alpha m{a}^{2}]\,\sin (ka)$$

5b $$B^{\prime} (k)=\frac{\partial B(k)}{\partial k}=8\,\alpha \beta [\cos (ka)-1][-\sin (ka)]$$

Equation (4) can be re-arranged as:

6 $$\frac{\partial \omega }{\partial k}[4Im{\omega }^{3}+2A(k)\omega ]=-\,[A^{\prime} (k){\omega }^{2}+B^{\prime} (k)]$$

or, more simply:

7 $$\frac{\partial \omega }{\partial k}=\frac{-[A^{\prime} (k){\omega }^{2}+B^{\prime} (k)]}{2\omega [2Im{\omega }^{2}+A(k)]}$$

In Eq. (7), it is worth noting that the angular frequency *ω* and the wavenumber *k* satisfy the wave dispersion relation shown in Eq. (1). Since we are considering the optical branch whose equation is shown in Eq. (3b), the *ω*^2^ term in Eq. (7) can be replaced as:

8 $$\frac{\partial \omega }{\partial k}=\frac{-[A^{\prime} (k){\omega }^{2}+B^{\prime} (k)]}{2\omega [-A(k)+\sqrt{A{(k)}^{2}-4ImB(k)}+A(k)]}=\frac{-[A^{\prime} (k){\omega }^{2}+B^{\prime} (k)]}{2\omega [\sqrt{A{(k)}^{2}-4ImB(k)}]}$$

In Eq. (8), the denominator is always positive for the optical branch. Therefore, the sign of ∂*ω*/∂*k* is governed by the sign of the numerator. The numerator in Eq. (8) can be expressed as:

9 $$-[A^{\prime} (k){\omega }^{2}+B^{\prime} (k)]=\,\sin (ka)\{[2(\alpha I+\beta m)-\frac{1}{2}\alpha m{a}^{2}]{\omega }^{2}+8\alpha \beta [\cos (ka)-1]\}$$

Due to the periodicity, the group velocities at *k* = 0 and *k* = *π*/*a* are already known to be zero, so we focus on the wavenumber range of 0 < *k* < *π*/*a*. At this range, sin (*ka*) is always positive. Thus, the sign of ∂*ω*/∂*k* is determined by its numerator as shown below:

10 $${\rm{sign}}(\partial \omega /\partial k)={\rm{sign}}\{[2(\alpha I+\beta m)-\frac{1}{2}\alpha m{a}^{2}]{\omega }^{2}+8\alpha \beta [\cos (ka)-1]\}$$

In Eq. (10), note that cos (*ka*) − 1 has a negative value at 0 < *k* < *π*/*a*. This indicates that 8*αβ* [cos (*ka*) − 1] has a negative value at 0 < *k* < *π*/*a*. Therefore, the sign of ∂*ω*/∂*k* depends on that of [2(*αI* + *βm*) − *αma*^2^/2]*ω*^2^ term. Because *ω*^2^ should be always positive, we can consider the following two cases:

11a $${\rm{CASE}}\,1:2(\alpha I+\beta m)-\,\frac{1}{2}\,\alpha m{a}^{2}\le 0,\,{\rm{i}}.\,{\rm{e}}.\,,\,\frac{I}{m}+\frac{\beta }{\alpha }\le \frac{{a}^{2}}{4}$$

11b $${\rm{CASE}}\,2:\,2(\alpha I+\beta m)-\frac{1}{2}\alpha m{a}^{2} > 0,\,{\rm{i}}.\,{\rm{e}}.\,,\,\frac{I}{m}+\frac{\beta }{\alpha } > \frac{{a}^{2}}{4}$$

In CASE 1, where the sum of the inertia ratio (*I*/*m*) and the stiffness ratio (*β*/*α*) is smaller than *a*^2^/4 (note that *a* is the periodicity), both 8*αβ* [cos (*ka*) − 1] and [2(*αI* + *βm*) − *αma*^2^/2]*ω*^2^ in Eq. (10) are negative at 0 < *k* < *π*/*a*. Therefore, in CASE 1, ∂*ω*/∂*k* is negative, and the optical branch has a negative group velocity as shown in Fig. 1(c).

In CASE 2, the group velocity ∂*ω*/∂*k* can be negative or positive. From Eq. (10), the corresponding conditions can be written as:

12a $$\frac{\partial \omega }{\partial k} > 0\,{\rm{if}}\,{\omega }^{2} > \frac{8\alpha \beta [1-\,\cos (ka)]}{2(\alpha I+\beta m)-0.5\,\alpha m{a}^{2}}$$

12b $$\frac{\partial \omega }{\partial k}=0\,{\rm{if}}\,{\omega }^{2}=\frac{8\alpha \beta [1-\,\cos (ka)]}{2(\alpha I+\beta m)-0.5\,\alpha m{a}^{2}}$$

12c $$\frac{\partial \omega }{\partial k} < 0\,{\rm{if}}\,{\omega }^{2} < \frac{8\alpha \beta [1-\,\cos (ka)]}{2(\alpha I+\beta m)-0.5\,\alpha m{a}^{2}}$$

**Figure 3:**
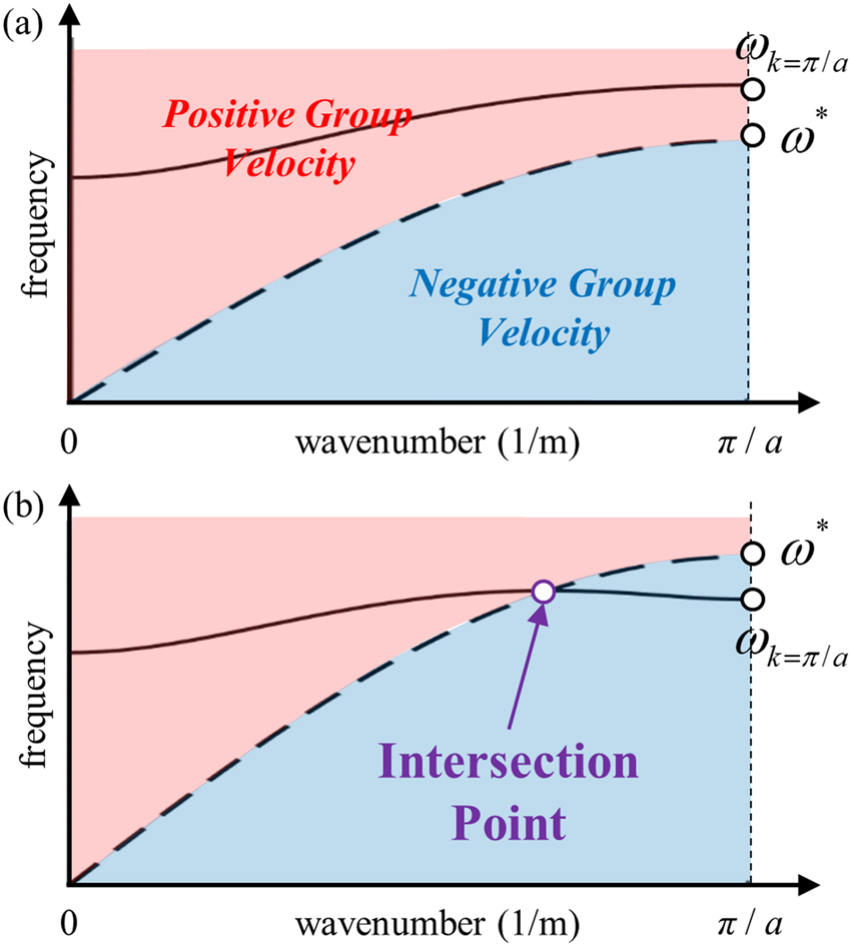
Plot of the wave dispersion curve (solid line) and Eq. (12b) (dashed line) when the optical branch has (**a**) purely positive group velocity and (**b**) both the positive-negative group velocities.

Again, we note that the angular frequency *ω* and the wavenumber *k* should satisfy the wave dispersion relation. Therefore, one may substitute the wave dispersion equation (shown in Eq. (3b)) with Eq. (12) to get the final condition. However, since this would be too complicated, we will use graphical approach instead. Figure 3 shows two regions satisfying Eqs (12a) and (12c), respectively. Here, the dotted line is the curve indicated in Eq. (12b). If the optical branch is in the red region, both the dispersion Eqs (3b) and (12a) are satisfied, and the group velocity ∂*ω*/∂*k* should thus be positive. On the other hand, if the optical branch is in the blue region, representing Eq. (12c), the group velocity ∂*ω*/∂*k* is negative. As illustrated by Fig. 3, if there is a point of intersection between the dotted line and the optical branch, i.e. if there exists a certain frequency *ω* that satisfies both Eqs (3b) and (12b), then the optical branch will exhibit both positive-negative group velocities (note that there can be only one intersection point – once the optical branch enters the blue region, the branch exhibits a negative slope and cannot intersect with the dotted line again). Also, it can be seen that when there is no intersection the optical branch exhibits a purely positive group velocity.

Thus, the condition of whether the group velocity exhibits purely positive group velocity or both the positive-negative group velocities can be established by the existence of the intersection point between the dotted line and the optical branch in Fig. 3(b). Since there can be at most one intersection point, we can focus on the frequencies of the optical branch at *k* = 0 and *k* = *π*/*a*. At *k* = 0, the dotted line is at zero frequency while the optical branch is located at nonzero frequency - the optical branch is thus in the red region at *k* = 0. Therefore, one may conclude that if the optical branch is in the blue region at *k* = *π*/*a*, i.e., if the dotted line has higher frequency than the optical branch at *k* = *π*/*a*, then there must exist an intersection point and thus the optical branch should have both positive-negative group velocities. Substituting *k* = *π*/*a* into Eqs (3b) and (12b) to figure out the frequencies of the optical branch yields:

13 $${\omega }_{k=\pi /a}^{2}=\frac{4\alpha }{m}\,({\rm{if}}\,\frac{I}{m} > \frac{\beta }{\alpha }),\,\frac{4\beta }{I}\,({\rm{if}}\,\frac{I}{m} < \frac{\beta }{\alpha })$$

14 $${\omega }^{\ast }=\frac{16\,\alpha \beta }{2(\alpha I+\beta m)-0.5\,\alpha m{a}^{2}}$$

while *ω*_*k*_ = _*π*/*a*_ is the frequency on the optical branch and ω^*^ is defined on the dotted line (see Fig. 3). From Fig. 3(a,b), it can be seen that there is no intersection point if *ω*_*k*_ = _*π*/*a*_ > ω^*^; otherwise, there is an intersection point and the optical branch exhibit both positive-negative group velocities. From these explanations, the following conditions can be achieved:purely positive group velocity if

15a $$\frac{4\alpha }{m}\ge {\omega }^{\ast }\,({\rm{if}}\,\frac{I}{m} > \frac{\beta }{\alpha })$$

15b $$\frac{4\beta }{I}\ge {\omega }^{\ast }\,({\rm{if}}\,\frac{I}{m} < \frac{\beta }{\alpha })$$

both positive-negative group velocities if

15c $$\frac{4\alpha }{m} < {\omega }^{\ast }\,({\rm{if}}\,\frac{I}{m} > \frac{\beta }{\alpha })$$

15d $$\frac{4\beta }{I} < {\omega }^{\ast }\,({\rm{if}}\,\frac{I}{m} < \frac{\beta }{\alpha })$$

Note that the denominator of Eq. (14) is positive in the CASE 2. Thus, Eq. (15) can be simplified by substituting Eq. (14) as:

purely positive group velocity if

16a $$\frac{I}{m}-\frac{\beta }{\alpha }\ge \frac{{a}^{2}}{4}\,({\rm{if}}\,\frac{I}{m} > \frac{\beta }{\alpha })$$

16b $$-\frac{I}{m}+\frac{\beta }{\alpha }\ge \frac{{a}^{2}}{4}\,({\rm{if}}\,\frac{I}{m} < \frac{\beta }{\alpha })$$

both positive-negative group velocities if

16c $$\frac{I}{m}-\frac{\beta }{\alpha } < \frac{{a}^{2}}{4}\,({\rm{if}}\,\frac{I}{m} > \frac{\beta }{\alpha })$$

16d $$-\frac{I}{m}+\frac{\beta }{\alpha } < \frac{{a}^{2}}{4}\,({\rm{if}}\,\frac{I}{m} > \frac{\beta }{\alpha })$$

**Figure 4:**
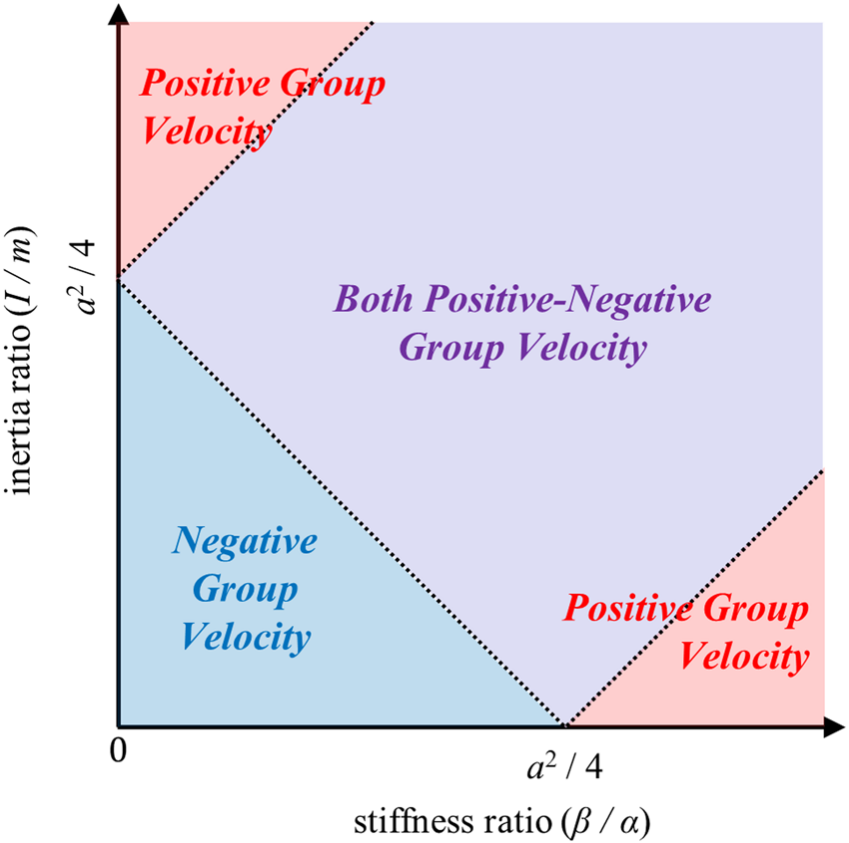
Visualized analytic condition for the group velocity in optical branch of flexural metamaterials.

Figure 4 summarizes the analytic conditions achieved in this section. As illustrated by Fig. 4, the group velocity of a flexural metamaterial is determined by its inertia ratio (*I*/*m*) and stiffness ratio (*β*/*α*). If the sum of two ratios is smaller than *a*^2^/4, the optical branch of the flexural metamaterial will have only negative group velocity. On the other hand, if the sum is larger than *a*^2^/4, the difference between two ratios plays important role. If the difference larger than *a*^2^/4, the optical branch will have a positive group velocity. Otherwise, both the positive and negative group velocities can be observed in the optical branch if the difference is smaller than *a*^2^/4. By manipulating these variables, one can tailor the flexural metamaterial to have a desired group velocity at its optical branch.

### Physical background of the exceptional group velocity issue

From the previous section, the analytic condition of positive, negative or positive-negative group velocities for the optical branch is derived. In this section, the physical origin of the group velocity issue is investigated. To identify the physical background, the real and imaginary parts of the wavenumber of the optical branch are focused. By re-arranging and solving the dispersion relation, in Eq. (1), with respect to wavenumber *k*, the following equations can be achieved. (see Supplementary Material for the detailed procedure):

17a $$k=\frac{1}{a}{\cos }^{-1}[\frac{-P(\omega )+8\,\alpha \beta -\sqrt{P{(\omega )}^{2}-16\,\alpha \beta Q(\omega )}}{8\,\alpha \beta }]$$

or

17b $$k=\frac{1}{a}{\cos }^{-1}[\frac{-P(\omega )+8\,\alpha \beta +\sqrt{P{(\omega )}^{2}-16\,\alpha \beta Q(\omega )}}{8\,\alpha \beta }]$$

**Figure 5:**
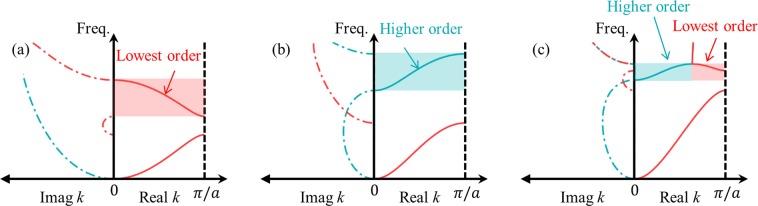
Plot of wave dispersion curves for both real and imaginary value of wavevectors. Here, flexural metamaterials whose optical branch has (**a**) purely negative group velocity, (**b**) purely positive group velocity and (**c**) both the positive-negative group velocities are considered, respectively.

According to Timoshenko beam theory, Eq. (17a) represents the wavenumber of the lowest order wave mode and Eq. (17b) represents the wavenumber of the higher order wave mode. From these two equations, dispersion curves can be plotted with both the real and imaginary wavenumber as shown in Fig. 5(a–c). The lowest order flexural wave mode is plotted in the red line and the higher order wave mode is plotted in the blue line. In addition, curves with the real wavenumber are plotted in the solid line while with the imaginary wavenumber are plotted in the dashed line.

First, consider the case in which the optical branch has a purely negative group velocity, whose dispersion curve is plotted in Fig. 5(a). As the figure shows, both the acoustic branch and optical branch consist of the red solid line, which means that both acoustic and optical branches belong to the lowest order flexural wave mode. For this case, the optical branch can be viewed as the band-folded wave of the lowest order flexural wave mode, which is generated by the periodicity. The higher-order flexural wave mode is shown to remain in the imaginary wavevector region, showing that the cutoff frequency of the higher-order flexural wave mode is higher than the optical branch.

Now, consider the case shown in Fig. 5(b) describing the case in which the optical branch has a purely positive group velocity. The acoustic branch is plotted in the red line while the optical branch is plotted in the blue line. At the frequency range of the optical branch, the lowest order flexural wave mode remains at the imaginary region with a real value of *k* = *π*/*a*. This indicates that the lowest order flexural wave mode is within the Bragg gap at the frequency range of the optical branch. Consequently, it may be concluded that the optical branch is the higher-order flexural wave mode while the acoustic branch is the lowest order flexural wave mode. In this case, the cutoff frequency of the higher-order flexural wave mode is very low such that the branch of the higher-order wave appears earlier than that of the band-folded lowest order flexural wave mode. Obviously, the branch should have a positive group velocity.

In Fig. 5(c), where the case in which the optical branch has both positive-negative group velocities is described, it can be seen that the optical branch is divided into two parts; the positive slope part (having a positive group velocity) is plotted in the blue line while the negative slope part (having a negative group velocity) is plotted in the red line. This indicates that the optical branch consists of both the lowest order flexural wave mode and the higher-order flexural wave mode. The higher-order flexural wave mode, having a positive group velocity, intersects with the band-folded lowest order flexural wave mode (a phenomenon usually called ‘branch-overlapping’), and both wave modes enter the imaginary plane at the intersection point. This is why the positive-negative group velocity is sometimes formed with the flexural metamaterial.

These results explain the background physics about how the flexural metamaterials can have negative, positive or even positive-negative group velocities. For other elastic wave mode such as longitudinal or shear waves, the higher-order wave modes have very high cutoff frequencies. Thus, there is generally no effect of higher-order wave mode on the optical branch for the longitudinal and shear wave modes. In flexural waves, however, the cutoff frequency of the higher-order wave modes is relatively low, and there can be possible interactions between the higher-order and the band-folded lowest-order wave modes. It results various abnormal group velocity phenomena in flexural metamaterials; the optical branch of flexural metamaterial can exhibit negative, positive or positive-negative group velocity.

### Numerical validation with wave dispersion curves

**Figure 6:**
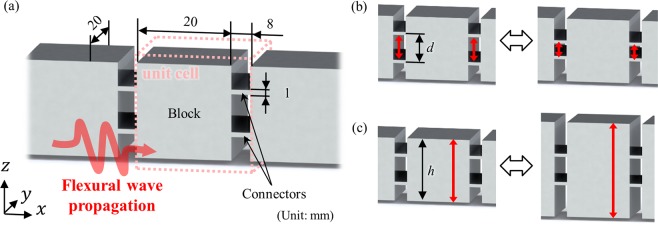
(**a**) Flexural metamaterial whose ratio and stiffness ratio can be easily tuned. Here, aluminum (E = 70 GPa, *v* = 0.31, *ρ* = 2700 kg/m^3^) is considered as the base material. (**b**) The stiffness ratio can be tuned by adjusting the distance *d* and (**c**) the inertia ratio can be tuned by adjusting the height *h*.

In the previous section it was shown that there is a certain condition defined by the inertia ratio and stiffness ratio, that determines whether the optical branch has positive, negative or both positive-negative group velocity in flexural metamaterials. To validate these analytic findings, the wave dispersion curves for various elastic metamaterials are numerically calculated. To effectively validate the analytic condition derived in the previous section, flexural metamaterials whose inertia ratio (*I*/*m*) and stiffness ratio (*β*/*α*) can be easily tuned should be introduced. To this end, the flexural metamaterial shown in Fig. 6(a) is considered. Figure 6(b,c) shows how the stiffness ratio and inertia ratio can be tuned with the proposed metamaterial; the stiffness ratio can be tuned by adjusting the distance between the connector couple *d*, while the inertia ratio can be tuned by changing the height of the block *h*. For instance, if the distance between the connector couple *d* increases, the vertical force generated by the connector is unchanged and the vertical linear spring *α* thus remains constant. However, increasing the distance *d* will increase the distance from the rotation center, which will increase the moment generated by the connector couple. Increasing the distance *d* thus increases the rotational spring *β*, and the stiffness ratio (*β*/*α*) can be adjusted by changing the distance between the connectors *d*. On the other hand, changing the thickness of the block *h* will both increase the mass *m* and the rotational inertia *I*. But the rotational inertia *I* is much more affected by the thickness *h* and thus *I* will be more increased. Accordingly, the mass ratio (*I*/*m*) can be easily tuned by changing the thickness of the block *h*.

**Figure 7:**
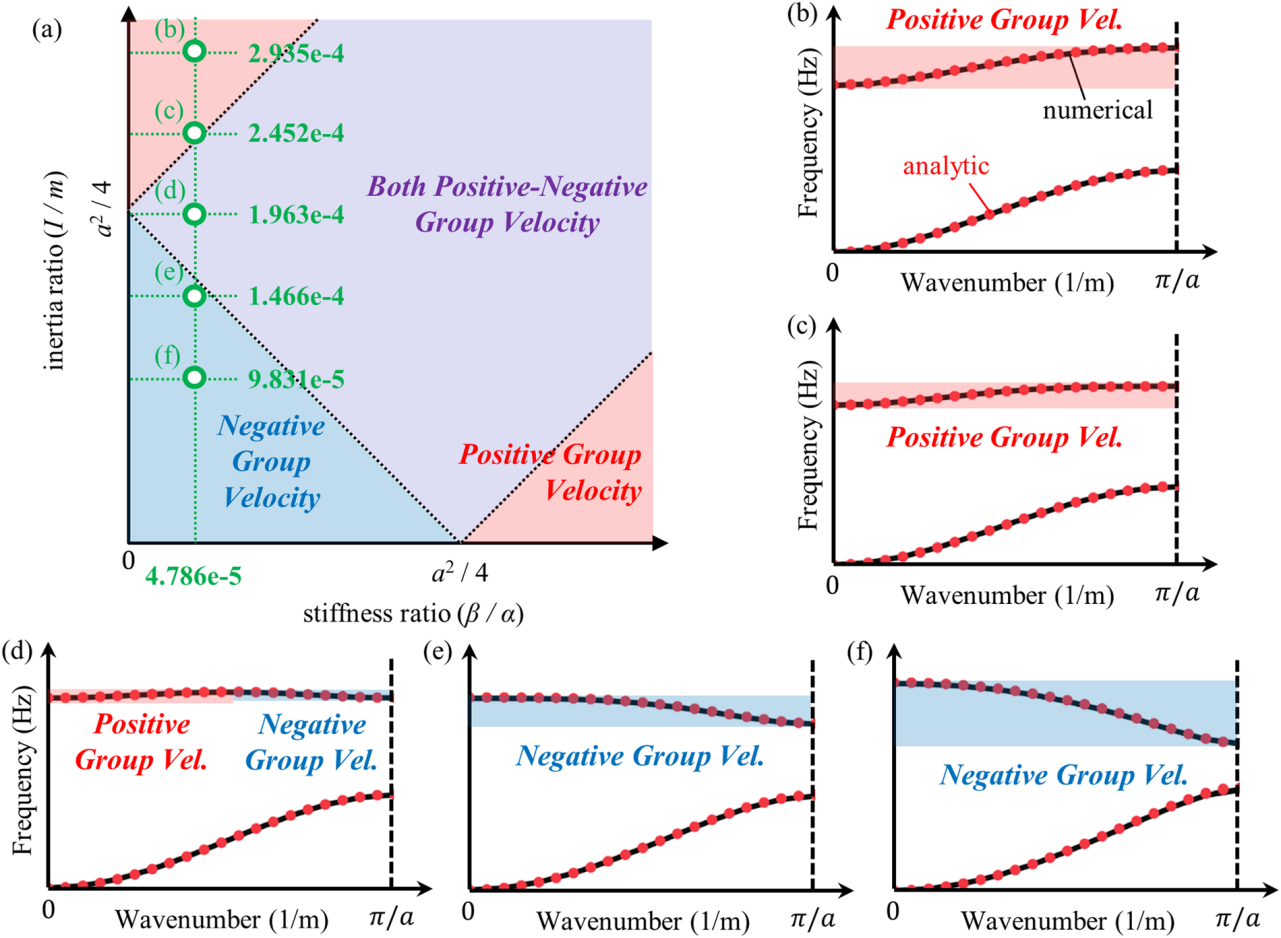
(**a**) Flexural metamaterials having constant stiffness ratio and various inertia ratio, and the wave dispersion curves for metamaterial with *d* = 0.7 mm and (**b**) *h* = 55.9 mm, (**c**) *h* = 50.4 mm, (**d**) *h* = 44.2 mm, (**e**) *h* = 36.9 mm and (**f**) *h* = 27.9 mm.

Based on the flexural metamaterial in Fig. 6(a), we conduct numerical validations for the analytic condition derived in Eq. (16). First, we considered 5 metamaterials having the same connector distance *d* but various thicknesses of block *h*, i.e., having the same stiffness ratio but different inertia ratio – the stiffness and inertia ratios of each metamaterial is plotted in Fig. 7(a). By using the numerical method with periodic boundary conditions^36^, the wave dispersion curve for the flexural elastic wave is numerically calculated for each metamaterial. For the comparison, the wave dispersion curve for each metamaterial is also analytically calculated by using Eq. (3). Here, coefficients, such as *α*, *β*, *m* and *I* for each metamaterials, are numerically calculated from static analyses as done in refs^10,11^. For instance, the equivalent vertical spring coefficient *α* is calculated by static numerical analysis imposing unit force along the thickness direction and measuring the corresponding displacement.

Figure 7(b–f) plots the numerically and analytically calculated wave dispersion curves. Note that in the numerical results, only the wave dispersion curve for the flexural wave mode is plotted; the curves for longitudinal and shear wave modes are omitted. Very good agreements are observed between numerical and analytic results, suggesting that the analytic approach used can well-predict both the actual wave behavior of the continuum flexural metamaterial and whether the optical branch of the metamaterial has positive, negative or both positive-negative group velocities. At the given stiffness ratio, the optical branch exhibits positive group velocities for large inertia ratios (see the metamaterial cases for Fig. 7(b,c)). However, as the inertia ratio decreases, the optical branch has both positive-negative group velocities as in Fig. 7(d) and, finally, it has a purely negative group velocity as in Fig. 7(e,f). This observation clearly validates the analytic condition summarized in Fig. 4.

**Figure 8:**
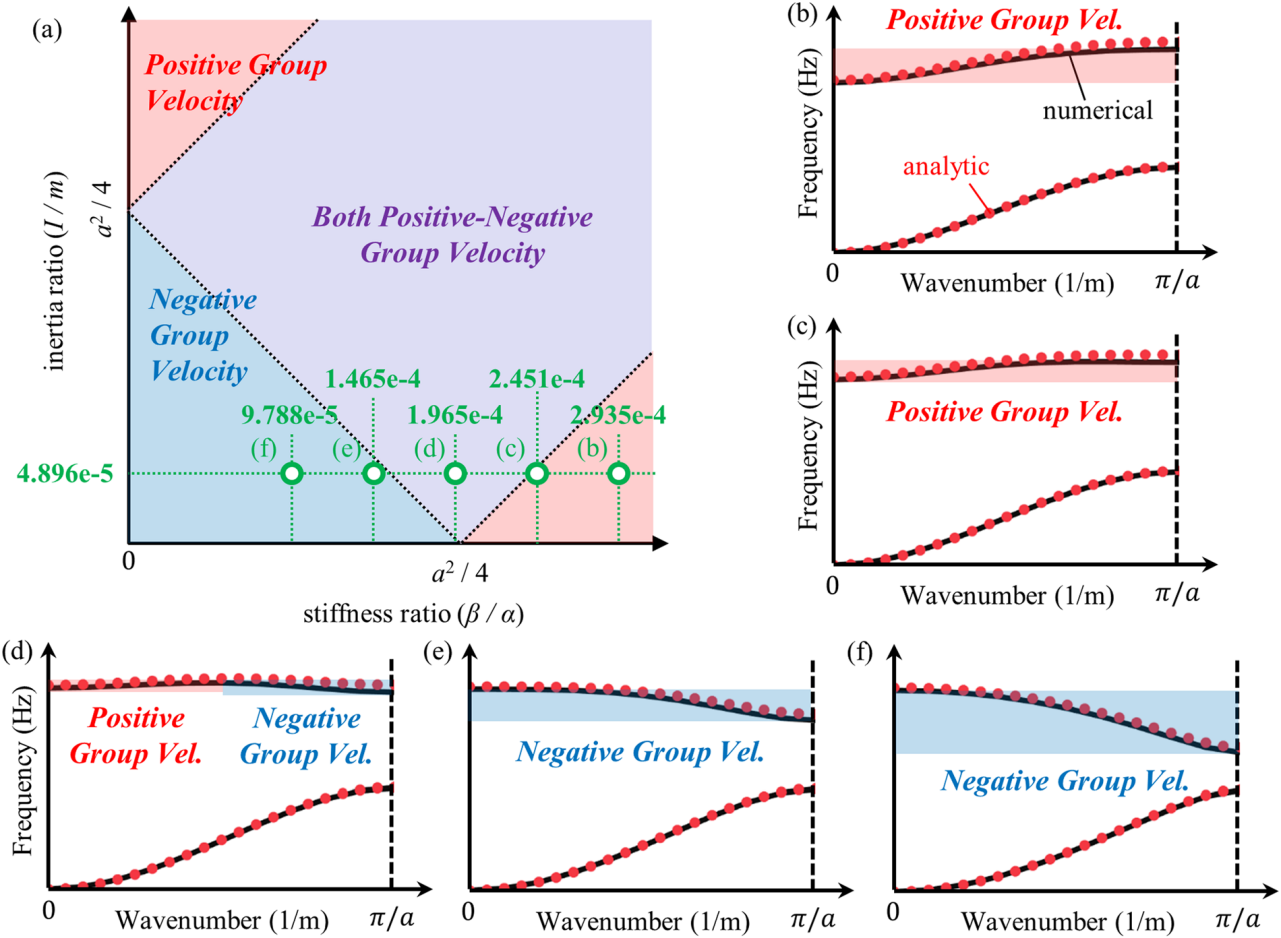
(**a**) Flexural metamaterials having constant inertia ratio and various stiffness ratio, and the wave dispersion curves for metamaterial with *h* = 13.7 mm and (**b**) *d* = 1.79 mm, (**c**) *d* = 1.58 mm, (**d**) *d* = 1.35 mm, (**e**) *d* = 1.08 mm and (**f**) *d* = 0.77 mm.

Now, we newly consider another 5 metamaterials with a constant inertia ratio but different stiffness ratio by adjusting the distance between the two connectors *d*. Figure 8(a) shows the stiffness and inertia ratio for each metamaterial. Again, the wave dispersion curves are both numerically and analytically calculated and plotted together in Fig. 8(b–f). From Fig. 8(b–f), one can deduce that the analytic condition derived in this work accurately predicts the group velocity of the metamaterial’s optical branch. For the constant inertia ratio, if the stiffness ratio is large as in Fig. 8(b,c), the group velocity of the optical branch is shown to be positive. However, the group velocity becomes both positive-negative and purely negative if the stiffness ratio decreases, as in Figs (d–f). This also supports the analytic condition summarized in Fig. 4. In conclusion, the numerical simulations clearly show that the sign of the group velocity of the flexural metamaterial’s optical branch is determined by the analytic condition derived in this work.

### Numerical validation with three-dimensional wave simulations

From the analytic investigations and numerical validations, it was shown that the optical branch of flexural metamaterials can have a negative, positive or both positive-negative group velocities according to its stiffness ratio and inertia ratio. However, the validation based on a wave dispersion curve may not provide clear results since the branch can be a ghost branch, deaf mode^37^ or other wave modes. Also, since all theories and dispersion analyses were made with two-dimensional structures, one may question that the results may not hold for three-dimensional metamaterial structures. Therefore, in order to further clarify the numerical validations, we carried out the negative and positive refraction simulation by using the three-dimensional flexural metamaterials, which are designed based on the analytic condition derived in this work.

**Figure 9:**
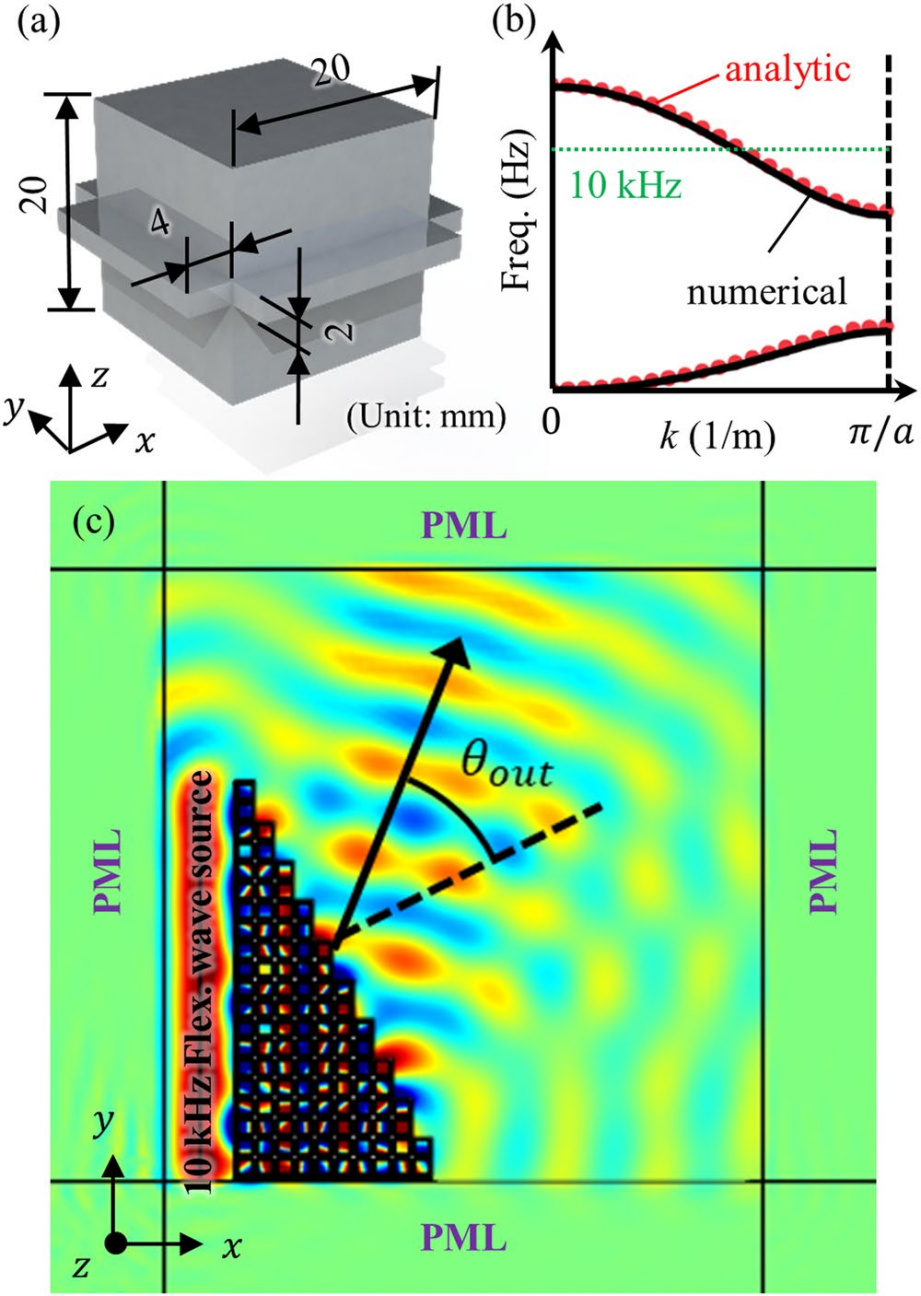
(**a**) Three-dimensional flexural metamaterial design having negative group velocity. (**b**) Wave dispersion curves achieved with numerical calculation (black solid line) and analytic equation (red dotted line). (**c**) Numerical wave simulation result at 10 kHz.

Figure 9(a) shows the three-dimensional unit cell, which is designed to have a negative group velocity. The metamaterial is designed to have a stiffness ratio of 6.667e-5 m^2^ and an inertia ratio of 8.268e-6 m^2^. According to the derived analytic condition, the metamaterial is expected to have a negative group velocity for its optical branch, as shown in Fig. 9(b). To show that the metamaterial indeed exhibits negative group velocity, a wave simulation is carried out. Here, the metamaterial is used to form a triangular prism, as in Fig. 9(c), and the flexural wave is actuated on the left side of the prism. Also, the perfectly matched layer (PML) is placed around the system to prevent any reflected waves from the boundary. Figure 9(c) shows the time-harmonic analysis results for the metamaterial shown in Fig. 9(a) (note that the simulation is a three-dimensional and the figure is plotted on the *x*-*y* plane). It can be seen that negative refraction phenomena can be observed from the simulation results – the refraction angle from the wave simulation is −41 degrees, which agree well with the analytically calculated refraction angle of −40.796 degrees. This result suggests that the analytic findings also hold for the three- dimensional case, and that the optical branch considered in this work is indeed the flexural wave, not the ghost branch, deaf mode or other wave modes.

**Figure 10:**
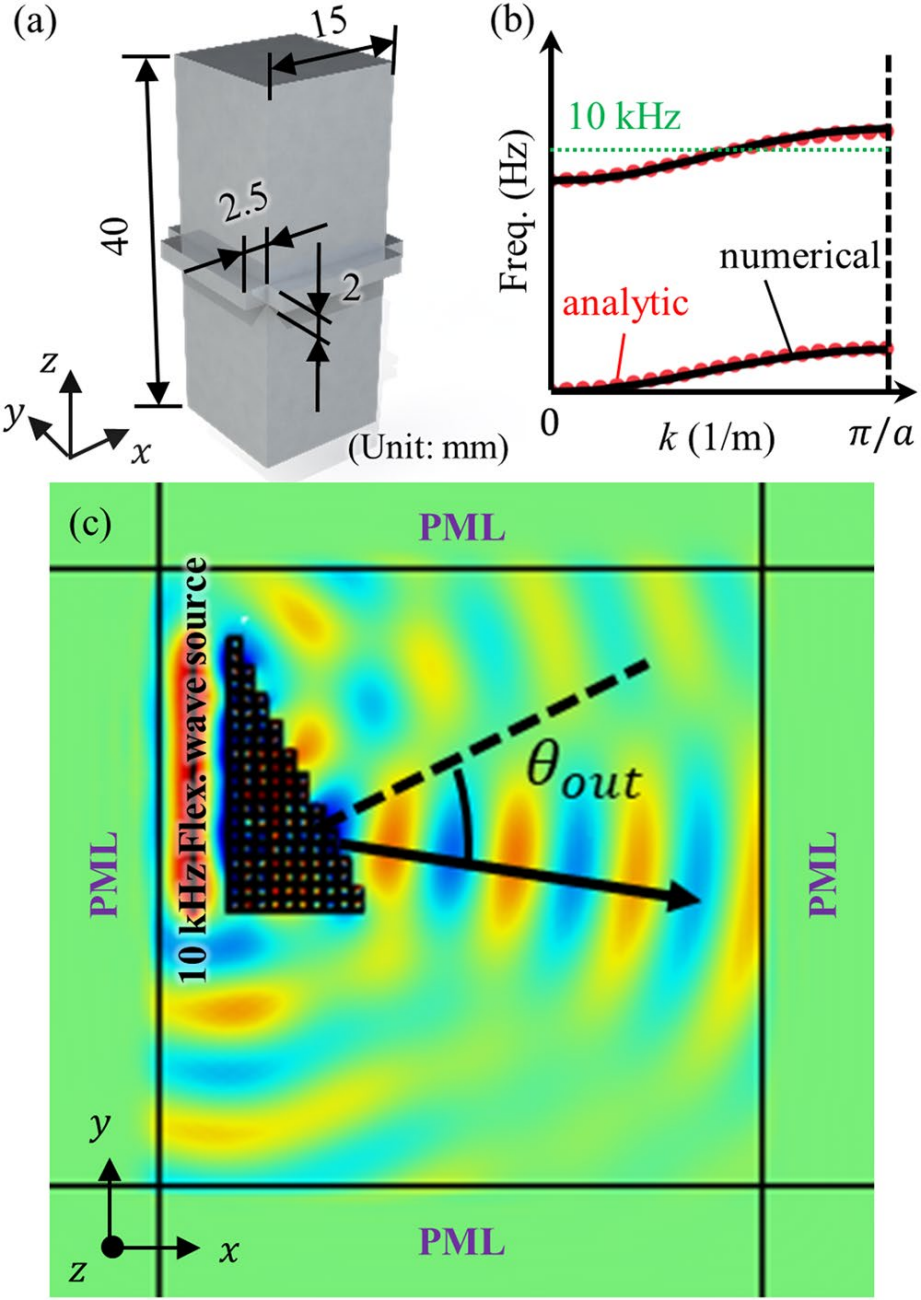
(**a**) Three-dimensional flexural metamaterial designed to have positive group velocity. (**b**) Wave dispersion curves achieved with numerical calculation (black solid line) and analytic equation (red dotted line). (**c**) Numerical wave simulation result at 10 kHz.

It was already shown that if the height of the block in the unit cell is increased, then the inertia ratio increases, and achieving a positive group velocity is possible. Figure 10(a) shows the three-dimensional unit cell which is designed in this way. With the unit cell, the stiffness ratio is measured to be 3.994e-6 m^2^ while the inertia ratio is evaluated as 1.521e-4 m^2^. These stiffness and inertia ratios are expected to provide the optical branch of the metamaterial having a positive group velocity, as shown in Fig. 10(b). To confirm this point, the same wave simulation is carried out by forming a metamaterial prism with the unit cell in Fig. 10(a). Figure 10(c) shows the wave simulation results. Here, the refraction angle is shown to be 38 degrees (from the wave simulation) and 39.857 degrees (from analytic calculations). The result shows that the metamaterials exhibit positive group velocity, validating the analytic investigations carried out in this work.

## Discussion

In this research, the abnormal group velocity generally observed in flexural metamaterial’s optical branches is analytically studied. Unlike other wave modes, the optical branch of the flexural metamaterial can be either band-folded, high-order branch or both, and the group velocity can be purely negative, positive or both positive-negative, respectively. To determine the detailed condition for the interaction, extended mass-spring systems for flexural metamaterial were considered. In the extended mass-spring system, both the vertical displacement and rotation of the metamaterial unit cell were considered to precisely analyze the wave motion in flexural metamaterials. From the analytic investigations, we found that the group velocity of the optical branch in flexural metamaterial is governed by a certain condition expressed by the stiffness and inertia ratio of the flexural metamaterial. Also, the analytic investigation showed that the abnormal characteristics originate from the higher-order flexural wave mode; flexural waves have very low cut-off frequency for high-order wave modes and thus the interaction between the band-folded lowest order flexural wave mode branch and high-order wave branch is highly active. If both the inertia and stiffness ratios are small, the optical branch of the flexural metamaterial is shown to be a purely band-folded lowest order flexural wave mode having negative group velocity. On the other hand, if one of the ratios is significantly larger than the other, the higher-order flexural wave mode dominates the optical branch and thus the optical branch exhibits purely positive group velocity. If both ratios are large and there is no significant difference between two ratios, the band-overlapping between the band-folded lowest order flexural wave mode and higher-order flexural wave mode takes place and thus the optical branch shows both positive-negative group velocities.

To validate the analytic investigation, numerical validations were carried out. In these validations, we introduced a new flexural metamaterial design whose stiffness and inertia ratios can be easily and independently tuned. Based on the design, flexural metamaterials having various stiffness and inertia ratios were numerically investigated by calculating their dispersion curves. Calculation results showed that the optical branch of the flexural metamaterial may have negative, positive or both positive-negative group velocity depends on the analytic condition derived. Also, additional wave simulation showed that the optical branch considered here is indeed the flexural wave’s dispersion branch. These results clearly validated the derived analytic condition.

We expect that the analytic condition derived in this paper will provide effective guidelines in designing various flexural metamaterials. In fact, although the issue related to the group velocity can be easily observed when designing flexural metamaterials, its physical background or any guidelines have not yet been given. By using the condition derived here, one can easily design metamaterials to have positive or negative group velocity by tailoring the stiffness and inertia ratios. Considering that flexural wave motion is closely related to vibrational applications, various new vibration systems, such as vibration-focusing devices, are expected to be newly designed based on the proposed analytic condition.

## Supplementary information

Supplementary Material

## Data Availability

The datasets generated during and/or analyzed during the current study are available from the corresponding author on reasonable request.

## References

[1] Fang N (2006). Ultrasonic metamaterials with negative modulus. Nat. Mater..

[2] Liu Y, Su X, Sun CT (2015). Broadband elastic metamaterial with single negativity by mimicking lattice systems. J. Mech. Phys. Solids.

[3] Huang HH, Sun CT (2011). Theoretical investigation of the behavior of an acoustic metamaterial with extreme Young’s modulus. J. Mech. Phys. Solids.

[4] Romero-García V, Krynkin A, Garcia-Raffi LM, Umnova O, Sánchez-Pérez JV (2013). Multi-resonant scatterers in sonic crystals: Locally multi-resonant acoustic metamaterial. J. Sound Vib..

[5] Hou Z, Assouar BM (2015). Tunable solid acoustic metamaterial with negative elastic modulus. Appl. Phys. Lett..

[6] Zhou X, Hu G (2009). Analytic model of elastic metamaterials with local resonances. Phys. Rev. B.

[7] Ding Y, Liu Z, Qiu C, Shi J (2007). Metamaterial with simultaneously negative bulk modulus and mass density. Phys. Rev. Lett..

[8] Huang HH, Sun CT, Huang GL (2009). On the negative effective mass density in acoustic metamaterials. Int. J. Eng. Sci..

[9] Zhu R, Liu XN, Hu GK, Sun CT, Huang GL (2014). A chiral elastic metamaterial beam for broadband vibration suppression. J. Sound Vib..

[10] Oh JH, Seung HM, Kim YY (2016). Adjoining of negative stiffness and negative density bands in an elastic metamaterial. Appl. Phys. Lett..

[11] Oh JH, Kwon YE, Lee HJ, Kim YY (2016). Elastic metamaterials for independent realization of negativity in density and stiffness. Sci. Rep..

[12] Khelif A, Choujaa A, Benchabane S, Djafari-Rouhani B, Laude V (2004). Guiding and bending of acoustic waves in highly confined phononic crystal waveguides. Appl. Phys. Lett..

[13] Khelif A, Djafari-Rouhani B, Vasseur JO, Deymier PA (2003). Transmission and dispersion relations of perfect and defect-containing waveguide structures in phononic band gap materials. Phys. Rev. B.

[14] Xiao Y, Wen J, Wen X (2012). Longitudinal wave band gaps in metamaterial-based elastic rods containing multi-degree-of-freedom resonators. New J. Phys..

[15] Sigalas M, Economou EN (1993). Band structure of elastic waves in two dimensional systems. Solid State Commun..

[16] Kushwaha MS, Halevi P, Dobrzynski L, Djafari-Rouhani B (1993). Acoustic band structure of periodic elastic composites. Phys. Rev. Lett..

[17] Oudich M, Li Y, Assouar BM, Hou Z (2010). A sonic band gap based on the locally resonant phononic plates with stubs. New J. Phys..

[18] Williams EG, Roux P, Rupin M, Kuperman WA (2015). Theory of multiresonant metamaterials for A0 Lamb waves. Phys. Rev. B.

[19] Assouar MB, Oudich M (2012). Enlargement of a locally resonant sonic band gap by using double-sides stubbed phononic plates. Appl. Phys. Lett..

[20] Yu D, Liu Y, Wang G, Zhao H, Qiu J (2006). Flexural vibration band gaps in Timoshenko beams with locally resonant structures. J. Appl. Phys..

[21] Pai PF, Peng H, Jiang S (2014). Acoustic metamaterial beams based on multi-frequency vibration absorbers. Int. J. Mech. Sci..

[22] Xiao Y, Wen J, Wen X (2012). Flexural wave band gaps in locally resonant thin plates with periodically attached spring–mass resonators. J. Phys. D. Appl. Phys..

[23] Pierre J, Boyko O, Belliard L, Vasseur JO, Bonello B (2010). Negative refraction of zero order flexural Lamb waves through a two-dimensional phononic crystal. Appl. Phys. Lett..

[24] Gusev VE, Wright OB (2014). Double-negative flexural acoustic metamaterial. New J. Phys..

[25] Lee SW, Oh JH (2018). Abnormal Stop Band Behavior Induced by Rotational Resonance in Flexural Metamaterial. Sci. Rep..

[26] Oh JH, Assouar B (2016). Quasi-static stop band with flexural metamaterial having zero rotational stiffness. Sci. Rep..

[27] Graff, K. F. *Wave Motion in Elastic Solids* Dover Publications, Inc (2012).

[28] Brillouin, L. *Wave propagation in periodic structures* Dover Publications, Inc (2003).

[29] Liu J, Hou Z, Fu X (2015). Negative refraction realized by band folding effect in resonator-based acoustic metamtaerials. Phys. Lett. A.

[30] Croenne C (2011). Negative refraction of longitudinal waves in a two-dimensional solid-solid phononic crystal. Phys. Rev. B.

[31] Lee MK, Ma PS, Lee IK, Kim HW, Kim YY (2011). Negative refraction experiments with guided shear-horizontal waves in thin phononic crystal plates. Appl. Phys. Lett..

[32] Zhu X, Zou X, Liang B, Cheng J (2010). One-way mode transmission in one-dimensional phononic crystal plates. J. Appl. Phys..

[33] Wu TT, Huang ZG, Tsai TC, Wu TC (2008). Evidence of complete band gap and resonances in a plate with periodic stubbed surface. Appl. Phys. Lett..

[34] Zhang X, Liu Z (2004). Negative refraction of acoustic waves in two-dimensional phononic crystals. Appl. Phys. Lett..

[35] Chiang C-Y, Luan P-G (2010). Imaging off-plane shear waves with a two-dimensional phononic crystal lens. J. Phys. Condens. Matter.

[36] Langlet P, Hladky-Hennion AC, Decarpigny JN (1995). Analysis of the propagation of plane acoustic waves in passive periodic materials using the finite element method. J. Acoust. Soc. Am..

[37] Hsiao FL (2007). Complete band gaps and deaf bands of triangular and honeycomb water-steel phononic crystals. J. Appl. Phys..

