# Supplementary material for: Study of Abnormal Group Velocities in Flexural Metamaterials
Source: Sci Rep. 2019 Sep 27;9:13973. doi: 10.1038/s41598-019-50146-8 (PMC6765006; doi:10.1038/s41598-019-50146-8)
Supplement: Supplementary file 1 — Supplementary Material [file 41598_2019_50146_MOESM1_ESM.pdf]

Supplementary Material

Study of Abnormal Group Velocities in Flexural Metamaterials

Hong Woo Park[1] and Joo Hwan Oh[1*]

[1]*School of Mechanical, Aerospace and Nuclear Engineering, Ulsan National Institute of Science and Technology, UNIST-gil 50, Eonyang-eup, Ulju-gun,Ulsan, 44919, Korea*

In this supplementary material, the detailed derivation of the dispersion relation for flexural waves in the extended mass-spring system shown in the main article is provided. Also, the detailed procedure for solving the dispersion relation with respect to the wavenumber to investigate both the real and imaginary wavenumber is explained.

* Corresponding Author, Assistant Professor, joohwan.oh@unist.ac.kr, phone +82-52-217-3051

Derivation of the dispersion relation for flexural wave in the extended mass-spring system

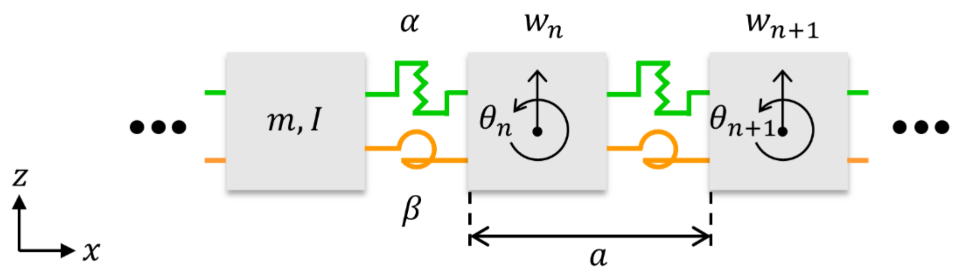

Figure S1. Extended mass-spring system for general flexural wave.

To consider the flexural wave which propagate in the general beam, the extended mass-spring system is considered as shown in Fig. S1. This mass-spring system consists of two kinds of springs (vertical linear spring α and rotational spring β) and two kinds of inertias (mass m and rotational inertia I) in order to consider vertical linear motion w_n and rotational motion θ_n of the nth unit cell of the system. The governing equations of motion for the nth unit cell can be expressed as:

$$m\frac{\partial^2 w_n}{\partial t^2} = \alpha\left(w_{n+1} - 0.5a\theta_{n+1} - w_n - 0.5a\theta_n\right) + \alpha\left(w_{n-1} + 0.5a\theta_{n-1} - w_n + 0.5a\theta_n\right), \qquad \text{(S1-a)}$$

$$\begin{aligned} I\frac{\partial^2 \theta_n}{\partial t^2} = \beta\left(\theta_{n+1} + \theta_{n-1} - 2\theta_n\right) + 0.5a\alpha\left(w_{n+1} - 0.5a\theta_{n+1} - w_n - 0.5a\theta_n\right) \\ - 0.5a\alpha\left(w_{n-1} + 0.5a\theta_{n-1} - w_n + 0.5a\theta_n\right). \end{aligned} \qquad \text{(S1-b)}$$

Where a denotes periodicity in Fig. S1. Since the system is periodic, we assume the periodic condition that the $n+1^{th}$ and $n-1^{th}$ terms can be converted into n^{th} term as shown below:

$$\begin{gathered} w_{n+1} = e^{-ika} w_n,\ w_{n-1} = e^{ika} w_n, \\ \theta_{n+1} = e^{-ika}\theta_n,\ \theta_{n-1} = e^{ika}\theta_n \end{gathered} \qquad \text{(S2)}$$

while k is the wavenumber along the propagating direction. By substituting equation (S2) to equation (S1) and assuming time harmonic motion with respect to the angular frequency of ω, equation (S1) can be re-written as:

$$-m\omega^2 w_n = \alpha\left(e^{-ika}w_n - 0.5ae^{-ika}\theta_n - w_n - 0.5a\theta_n\right) + \alpha\left(e^{ika}w_n + 0.5ae^{ika}\theta_n - w_n + 0.5a\theta_n\right) \quad \text{(S3-a)}$$

$$\begin{aligned}-I\omega^2\theta_n = {} & \beta\left(e^{-ika}\theta_n + e^{ika}\theta_n - 2\theta_n\right) + 0.5a\alpha\left(e^{-ika}w_n - 0.5ae^{-ika}\theta_n - w_n - 0.5a\theta_n\right) \\ & - 0.5a\alpha\left(e^{ika}w_n + 0.5ae^{ika}\theta_n - w_n + 0.5a\theta_n\right)\end{aligned} \quad \text{(S3-b)}$$

Equation (S3) can be simplified as:

$$-m\omega^2 w_n = \alpha\left(e^{-ika} + e^{ika} - 2\right)w_n + 0.5\alpha a\left(e^{ika} - e^{-ika}\right)\theta_n \quad \text{(S4-a)}$$

$$-I\omega^2\theta_n = \beta\left(e^{-ika} + e^{ika} - 2\right)\theta_n + 0.5a\alpha\left(e^{-ika} - e^{ika}\right)w_n - \left(0.5a\right)^2\alpha\left(e^{ika} + e^{-ika} + 2\right)\theta_n \quad \text{(S4-b)}$$

By applying Euler's equation as $e^{-ika} + e^{ika} = 2\cos(ka)$ and $e^{ika} - e^{-ika} = i2\sin(ka)$, equation (S4) can be expressed as:

$$-m\omega^2 w_n = 2\alpha\left(\cos(ka) - 1\right)w_n + i\alpha a\sin(ka)\theta_n \quad \text{(S5-a)}$$

$$-I\omega^2\theta_n = -ia\alpha\sin(ka)w_n + \left\{2\beta\left[\cos(ka) - 1\right] - 0.5a^2\alpha\left[\cos(ka) + 1\right]\right\}\theta_n \quad \text{(S5-b)}$$

Expressing equation (S5) in matrix form yields:

$$\begin{bmatrix} 2\alpha\left(\cos(ka) - 1\right) + m\omega^2 & i\alpha a\sin(ka) \\ -i\alpha a\sin(ka) & 2\beta\left[\cos(ka) - 1\right] - \frac{1}{2}a^2\alpha\left[\cos(ka) + 1\right] + I\omega^2 \end{bmatrix}\begin{bmatrix} w_n \\ \theta_n \end{bmatrix} = \begin{bmatrix} 0 \\ 0 \end{bmatrix} \quad \text{(S6)}$$

To avoid any trivial solution for equation (S6), determinant of the matrix should be zero. As a result, the dispersion relation for flexural wave in the extended mass-spring system can be derived as:

$$Im\omega^4 + A(k)\omega^2 + B(k) = 0 \quad \text{(S7)}$$

where $A(k)$ and $B(k)$ are defined as:

$$A(k) = 2(\alpha I + \beta m)\left[\cos(ka) - 1\right] - \frac{1}{2}\alpha m a^2\left[\cos(ka) + 1\right] \quad \text{(S8-a)}$$

$$B(k) = 4\alpha\beta\left[\cos(ka) - 1\right]^2 \quad \text{(S8-b)}$$

Physical background of the exceptional group velocity issue

To solve equation (S7) with respect to wavenumber k, re-arranging the dispersion relation in equation (1) with $X = \cos(ka) - 1$ results in:

$$4\alpha\beta X^2 + P(\omega)X + Q(\omega) = 0, \quad \text{(S9)}$$

Where:

$$P(\omega) = \left[2(\alpha I + \beta m) - 0.5\alpha m a^2\right]\omega^2 \quad \text{(S10-a)}$$

$$Q(k) = Im\omega^4 - \alpha m a^2 \omega^2. \quad \text{(S10-b)}$$

By using the quadratic formula, the solution for equation (S9) can be obtained as:

$$X = \frac{-P(\omega) + \sqrt{P(\omega)^2 - 16\alpha\beta Q(\omega)}}{8\alpha\beta} \quad \text{or} \quad \text{(S11-a)}$$

$$X = \frac{-P(\omega) - \sqrt{P(\omega)^2 - 16\alpha\beta Q(\omega)}}{8\alpha\beta} \quad \text{(S11-b)}$$

Since $X = \cos(ka) - 1$, equation (S11) can be expressed as:

$$k = \frac{1}{a}\cos^{-1}\left[\frac{-P(\omega) + 8\alpha\beta + \sqrt{P(\omega)^2 - 16\alpha\beta Q(\omega)}}{8\alpha\beta}\right] \quad \text{or} \quad \text{(S12-a)}$$

$$k = \frac{1}{a}\cos^{-1}\left[\frac{-P(\omega) + 8\alpha\beta - \sqrt{P(\omega)^2 - 16\alpha\beta Q(\omega)}}{8\alpha\beta}\right] \quad \text{(S12-b)}$$

According to classical Timoshenko beam theory[S1], equation (S12-a) represents the wavenumber of the lowest order flexural wave mode and equation (S12-b) indicates the wavenumber of the higher-order flexural wave mode. From these two equations, both the real and imaginary parts of wavenumbers are derived, and the physical origin of each branch can be investigated as in the manuscript.

References

[S1] Graff, K. F. *Wave Motion in Elastic Solids*. Dover Publications, Inc (2012).
